# ‘I wouldn’t say it’s sexism, except that … It’s all these little subtle things’: Healthcare scientists’ accounts of gender in healthcare science laboratories

**DOI:** 10.1177/0306312712460606

**Published:** 2013-02

**Authors:** Valerie Bevan, Mark Learmonth

**Affiliations:** Department of Management Learning and Leadership, Lancaster University Management School, Lancaster University, Lancaster, UK; Durham Business School, Durham University, Durham, UK

**Keywords:** feminist research, healthcare science, taken-for-granted masculinities, under-representation of women, women in science

## Abstract

We explore healthcare scientists’ accounts of men in healthcare science laboratories. By focussing on subtle masculinist actions that women find disadvantageous to them, we seek to extend knowledge about women’s under-representation in senior positions in healthcare science – despite women being in the majority at junior levels. We maintain that healthcare science continues to be dominated by taken-for-granted masculinities that marginalize women, keeping them in their ‘place’. Our aim is to make visible the subtle practices that are normally invisible by showing masculinities in action. Principally using feminist analyses, our findings show that both women and men are often unaware of taken-for-granted masculinist actions, and even when women do notice, they rarely challenge the subtle sexist behaviour.

## Introduction

Why are women seriously under-represented in senior positions within UK healthcare science?^1^ The puzzle is particularly striking because women healthcare scientists^2^ are in the majority – in stark contrast with science, engineering and technology (SET) professions and associated professions generally, where women represent only 15.5 percent of all scientists (Kircup et al., 2010: 74). National figures are not available, but the major employer of healthcare scientists in which this study was largely based (called ‘PQR’ for the purposes of this article) employs 1600 healthcare scientists, of whom 60 percent are women. However, all 13 healthcare scientists in the most senior grade are men, as are more than two-thirds of the 110 staff in the two grades below. A similar pattern exists in academic posts in biosciences more generally where comparable numbers of women and men gain PhDs, but fewer than 10 percent of professors in biosciences are women (Kircup et al., 2010: 60).

In this article, we seek to contribute to an understanding of the marginalization of women that leads to their under-representation in senior posts in healthcare science, an analysis that will be of relevance to scientists more generally. In doing so, we especially seek to challenge the failure highlighted in much of the mainstream literature on scientific life:

to take serious notice not only of the fact that science has been produced by a sub-set of the human race – that is, almost entirely by white, middle-class men – but also of the fact that it has evolved under the formative influence of a particular ideal of masculinity [associated with] ‘virile’ power. (Keller, 1985: 7)

We are also concerned, therefore, to challenge the gendered division of labour more generally in healthcare and other sciences. We note, for example, women’s lower pay overall, and the lack of esteem for those scientists (overwhelmingly women) who work on part-time or on temporary, contracts (Crompton and Lyonette, 2011; Valian, 2004); we also note that salary progression and gaining tenure happen more slowly for women (Valian, 2000).

To address these challenges, we present readings of interviews with UK-based healthcare scientists conducted between 2006 and 2008.^3^ Our readings are particularly influenced by traditions within feminist research concerned with the apparently mundane and taken-for-granted discursive construction of gendered differences: ‘the ways in which power is relayed in everyday practices … [in the] minutiae of social relations … [and] through seemingly trivial incidents and transactions’ (Morley, 2006: 543).

We are also informed by the experiences that one of us has had from a lifetime’s career as a healthcare scientist herself. The first author started work as a laboratory technician in the 1960s and quickly learned her ‘place’ (Miller, 1986: 75; Newman, 1995: 19) in the hierarchy of the healthcare science laboratory – an environment dominated by men. This environment was similar to that described by Kemelgor and Etzkowitz (2001) in academic science: where women face ‘ongoing subtle and overt exclusion’ (p. 240), where ‘two worlds’ (p. 242) exist, one for men and another for women, and where women are denied access to communication and support mechanisms that are available to men. However, although always perturbed by such difficulties, she had no language through which to question and oppose – or even fully articulate – these difficulties and ‘assum[ed she was] … deficient’ (Miller, 1986: xiii).

Like the women interviewed, the first author rarely challenged the masculinist actions for much of her working life. This began to change around 2000, upon taking a part-time master’s degree in management studies, when, for the first time, she became exposed to feminist concepts that had previously been (at best) on the periphery of her consciousness. She then started to see ways to articulate better the sorts of concerns she had long been bothered by but had not been able properly to name. Indeed, enthusiasm for more feminist ideas was such that she continued to study part-time until 2009, gaining a social science PhD, while working full-time in PQR. Hence, we wish to emphasize that we are seeking to be politically engaged in this article – to produce ‘passionate scholarship’ (Dubois, 1983: 108) – because of our personal stakes in the issues studied.

However, readings that emphasize the subtle nature of women’s disadvantage in healthcare science are offered, not merely because they resonate with the experiences one of us has had as a woman working in healthcare science, but also because arguments of this nature are underplayed in many of the official, policy-orientated reports intended to improve the numbers of senior women in UK healthcare science. Subtle forms of discrimination have become the commonest sort of explanation for the under-representation of women in science (Rhoton, 2011) and other similarly prestigious occupations, at least among social scientists (Bendl, 2008; Benokraitis and Feagin, 1995; Fotaki, 2011; Jeanes et al., 2011; Morley, 2006; Pringle, 1998; Risman, 2004; Wajcman, 1998; and see specialist journals including, for example, *Gender & Society* and *Gender, Work & Organization*). However, any kind of research of this nature appears to have been more or less unknown to the scientists interviewed in our study, participants (both women and men) still appearing to favour theories of women’s ‘lack’ to explain gendered inequality.

We speculate that the novelty of our work for many healthcare scientists may arise in part from the hegemonic status still accorded to the experimental paradigm and the idea that ‘science values control and subordination’ (Martin, 2001: 608; see also Albert et al., 2008). While experimental methods are clearly appropriate for the conduct of healthcare science *as science*, its hegemony is problematic nevertheless because it appears to have led to a disregard for interpretive social scientific research in the study of the social practices within healthcare science. As Albert et al. (2008: 2529) observe ‘our findings suggest that social scientists who pursue qualitative methodologies currently have the support of only a subset of biomedical scientists, mainly those who have been exposed to social science research.’ We use qualitative methods influenced by feminist ideas in the belief that they represent a means to explore issues that, without their use, are difficult to see, or even to name.

Our aims, then, are twofold. The first aim is to highlight how our findings in healthcare science share affinities with, but also significant differences from work using similar methods and assumptions conducted in other areas of science. In other words, we aim to contribute to knowledge about gendered relations in healthcare science and in science more generally. Our second aim is complementary to the first. It is to use our research as a vehicle to suggest how things might be changed in science – especially, though not exclusively, in healthcare science.

This article proceeds as follows: First, we review some of the key findings about the position of women in science and how these findings have been used (or not) in relation to current policymaking concerning gender equality in healthcare science. After an account of methodology, we turn to the empirical materials. Following further discussion, we conclude with suggestions about how feminist ideas, while hardly guaranteeing change, have the potential to make a positive difference to the situations of women and men in healthcare science.

## Women and the masculinist discourse in healthcare science workplaces

Science, a hugely influential and prestigious domain still overwhelmingly dominated by men, has long represented a target for feminist analyses (Creager et al., 2001; Harding, 1991; Keller and Longino, 1996; Schiebinger, 1989). Of course, there are varieties of feminisms (Calás and Smircich, 1996; Code, 2000), just as there are multiple aspects of science that have attracted feminists’ interest. But in this article we focus on feminist work that draws attention to the more subtly damaging masculinist discourse that affects the taken-for-granted ‘patterns of behaviour, beliefs, symbols and identity reproduced’ within organizations (Hearn, 2002: 42). This masculinist discourse is the foundation for the subtle sex discrimination that comprises ‘the unequal and harmful treatment of women that is typically less visible … [and] is often not noticed because most people have internalized subtle sexist behaviour as “normal”, “natural”, or customary’ (Benokraitis and Feagin, 1995: 41). Furthermore, because it is generally unnoticed, the perpetrators (and the victims) may not be fully aware of its effects.

The sort of subtle, discursive enactments of gender advantage to which we draw attention have been subjected to analysis by social scientists in fields outside healthcare science, including in broader science itself (Creager et al., 2001; Harding, 1991; Keller and Longino, 1996; Schiebinger, 1989; Sonnert and Holton, 1995), in academia (Husu, 2001; Kantola, 2008; Morley, 1999, 2006), in health (Witz, 1988, 1992), in nursing (McMurray, 2011) and in medicine (Crompton and Lyonette, 2011; Pringle, 1998). We have also been influenced by similar work in organization studies (Bendl, 2008; Benokraitis and Feagin, 1995; Ford and Harding, 2010; Ford et al., 2012; Katila and Merilainen, 1999; Learmonth and Humphreys, 2012; Martin, 2001) and management and leadership more specifically (Billing, 2011; Broadbridge and Hearn, 2008; Page, 2011). While not all these analyses are discursive in orientation, they do show that subtle forms of sexism are recognized widely in social science publications. Indeed, in the discussion of female gender disadvantage in science itself, Valian (2004) coined the term ‘gender schemas’ to describe the subtle actions that work to ‘disadvantage’ women (p. 208), suggesting that in science:

[t]he main answer to the question why there are not more women at the top is that our gender schemas skew our perceptions and evaluations of men and women, causing us to overrate men and underrate women. Gender schemas affect our judgments of people’s competence, ability and worth. (p. 208)

Referring to the work of Acker (1990) and Martin (2001) among others, Rhoton (2011) also describes how these subtle ‘gendered structures and cultures impose sets of masculinized expectations on scientists that limit the range of acceptable behaviours and professional demeanours’ (pp. 696–697) open to senior female academic scientists. Rhoton (2011) concludes that ‘many of the harmful practices in which men engage may not be intentional’ but, of particular note to us, that women scientists themselves ‘may also contribute to gendered barriers’ (p. 697). Women, Rhoton (2011) argues, contribute to the gendered practices because they do not distinguish them from ‘normal’ scientific professional practices that they see as parts of ‘gender-neutral meritocracies’ (p. 699). In Rhoton’s (2011) interpretation, women do not want to emphasize their difference but rather want to ‘fit in’ (p. 706); they eschew practices they regard as feminine or as associated with women because these tend to perpetuate female stereotypes such as ‘women do not take criticism … well’ (p. 702). In science, then, femininity is ‘constructed as subordinate to masculinity’ (Rhoton, 2011: 707, citing West and Zimmerman, 1987). And, as Fotaki (2011) reports, in academe, women’s ‘embodied (sexual) presence largely determined what they could (not) do or say at work and the position from which they could (not) do so’ (p. 45).

Thus, as Fletcher (2001) describes, when women manifest ‘empathy, vulnerability and connection’ (p. 9), they are regarded as too caring or relational. Carlson and Crawford (2011) go a stage further and suggest that relational practices are regarded as ‘ineffective’ (p. 371) whether they are performed by women or by men; such practices are rarely rewarded and are even actively discouraged by both sexes partly because they do not have the language to describe them in ways acceptable in organizational life (p. 363). We suggest that assessing women as caring and therefore ineffective provides a rationale for women to be excluded from decision-making groups.

Of course, the lack of women’s progress in science has been recognized among the UK science official policymaking bodies – albeit not in explicitly feminist terms. For example, a pivotal report advised the UK government in 2002 on improving the education and employment of women in science and related disciplines (Greenfield et al., 2002). Emphasizing, in particular, the business advantages of promoting equality, the Greenfield Report was highly critical of the current situation and stated that the ‘problem … cannot be tackled with one sweeping action but … requires a pragmatic and consistent approach from the organizations involved in managing and nurturing the nations’ scientists’ (Greenfield et al., 2002: 9). As part of its acceptance of the Greenfield Report, the Department of Trade and Industry (DTI, 2003) set up the UK Resource Centre for Women (UKRC) in SET with the aim of delivering the government’s equality strategy. Indeed, in fulfilment of this role, the UKRC’s recent guide (Kircup et al., 2010) promotes gender equality in science organizations, in which it continues to emphasize the business advantages of gender equality in ways established by the Greenfield Report. These are advantages, which, they hold, lead to ‘better business performance’ (Kircup et al., 2010: 6).

These policy reports do refer to a limited amount of research similar to our own. For example, noting the work of Morley, Greenfield et al. (2002) suggest that women in science tend not to ‘complain of overt discrimination but of having to work harder to convince and persuade their managers that they want and need more responsibility which they see being given automatically to their male colleagues’ (p. 45).

Greenfield et al. (2002) also include a brief discussion of the factors exacerbating attrition, which they state includes the ‘[l]ack of transparency of appointments process … sexual harassment/bullying/continual teasing – often subtle and even unintentional’ (p. 46). While we welcome these official acknowledgements of some of the problems, we feel, nevertheless, that such initiatives have yet to recognize sufficiently the masculinist discourse that underlies much scientific practice. In particular, the emphasis by Greenfield et al. on the business case for gender equality, endorsed by Kirkup et al. (2010), who state that gender equality is a ‘business bottom-line issue’ (p. 34), arguably reinforces this masculine culture. We follow Perriton (2009) in arguing that ‘[t]he standard business case is problematic because it constrains, rather than opens up, the discussion of social justice issues in the workplace’ (p. 240).

Our insights are offered to policymakers, therefore, because the emphasis on women’s accounts of their experiences brings new insights into why women remain predominantly in the lower grades and are hindered from reaching their potential. Various other initiatives at the UK level,^4^ European level^5^ and in the Unites States^6^ are working to improve the employment practices that currently hinder women’s advancement, but these schemes seem to be mainly focussed on the case for improving research science rather than public sector healthcare science, and not focussed on the subtle disadvantages women face. Nevertheless, there could be lessons to be learnt for healthcare science.

Thus, our analyses address gender issues discursively, in significantly greater detail than found in these reports, within an explicitly feminist framework and in the specific context of healthcare science. Our particular contribution is to provide rich descriptions of the subtly gendered phenomena that women in healthcare science interpret as harmful to them. These descriptions are offered as potentially valuable both to individual scientists and to policymakers. So, let us turn to our methods.

## Methodology

Our study is based upon 42 in-depth interviews conducted by the first author. I (the first author) therefore describe my work in the first person singular in this section. The first-person methods account is a deliberate rhetorical trope, intended, in part, to distance me from the ‘scientific’ (and masculinized) convention of a supposedly impersonal third-person passive voice (Martin, 1990; Watson, 1995). But I also use it in order to emphasize that my personal identity, as the interviewer, is, in itself, an important factor in the overall research process. As Ramazanoğlu and Holland (2002) remind us, ‘feminist methodology cannot be independent of the ontology, epistemology, subjectivity politics, ethics and social situation of the researcher’ (p. 16).

The majority of the people I interviewed (38/42) were healthcare scientists, 35 of whom worked in the public sector and three in private sector laboratories related to healthcare. The four (of 42) people interviewed who were not healthcare scientists were chief executives or Board members, interviewed because they occupied key positions of influence in public scientific institutions. The majority (33/42) were employed within PQR, a pseudonym for a large publicly funded body outside the National Health Service (NHS) and concerned with delivering health-related laboratory services, among other things. I interviewed a wide range of healthcare scientists – from the most senior managers to those carrying out day-to-day laboratory work. They came from ethnically diverse backgrounds. Although my primary interest was in the accounts of women in science, I interviewed 11 men, from a conviction that men’s views and experiences were likely to be important for women (De Cheveigné, 2009; Gatrell, 2006; Stanley and Wise, 1993), given the traditionally male-dominated nature of laboratories, where men fill the majority of the senior posts despite being in the numerical minority in the healthcare science workforce overall.

Interviews typically lasted 60–90 minutes, each one being taped and transcribed in full. The complete set of transcriptions totalled some 431,200 words. All participants knew that I was interested in why women do not progress in science to the same degree as their male counterparts. I invited participants to talk broadly about their work-based experiences and, in keeping with Martin’s (2001) consideration that ‘announcing a focus on gender prompted defensiveness [and] confusion’ (p. 594), I was generally able to prompt them to discuss how they thought their gender had affected their careers in science. I did not express any specific feminist ideas, as I considered this might be counterproductive in an environment where feminism has a low profile and a ‘negative reputation’ (Ramazanoğlu and Holland, 2002: 157).^7^

My overall approach, then, was heavily influenced by authors who have outlined feminist interview methodologies (Oakley, 1981; Ramazanoğlu and Holland, 2002; Stanley and Wise, 1993), and so I adopted a style of interviewing that was conversational, open and empathetic. I self-consciously steered away from the (masculinized) ‘proper’ interview, described by Oakley (1981) as one where ‘[t]he motif of successful interviewing is “be friendly but not too friendly”’ (p. 33), allowing our conversations to flow in the directions that emerged. Most of the interviewees, both men and women, seemed to enjoy talking about themselves and having the chance to be reflective, apparently rarely having such an opportunity. Such two-way sharing was assisted by being professionally acquainted with 28 of the interviewees; the others were either part of the same organization or had been recommended to me. As a healthcare scientist myself, with a career of over 40 years in UK public sector laboratories, my background was similar to those of several of the women interviewees and I felt that this understanding facilitated the interview discussions.

I analysed the interview transcripts together with the second author. We felt that joint analysis was particularly important, as working together enabled us to view the empirical material from a number of standpoints. I interpreted the accounts of women and men from a position reliant upon my years of experience as a female healthcare scientist. My colleague, a male social scientist and pro-feminist – someone with a ‘commitment to gender equality as part of contemporary modern masculinity’ (Bjørnholt, 2011: 6; see also Hearn, 2000) – has never worked in a laboratory, but could bring to the analysis his experience of being a senior man in a university, along with his awareness of (some of!) the advantages of being a (white, middle-class, heterosexual) man. Indeed, working on the analysis has brought home to him new ways in which some of his own actions at work might have been harmful to women.

Our analysis has been developed primarily with reference to the women’s accounts. But we have also included a number of the men’s accounts of similar situations (with their usually rather different interpretations), with the men’s interpretations being read in the light of the women’s accounts. However, this interpretive strategy should not be taken to imply that we think women’s accounts are inherently ‘better’ than men’s. Rather, we focus on women’s accounts both because they are often significantly different from men’s and also because such subordinate voices are rarely heard within healthcare science. Indeed, we follow Miller (1986) in believing that:

The dominant-subordinate situation was – and is – depriving and distorting to members of both sexes, but in different ways for each. The point is that the close study of an oppressed group reveals that a dominant group inevitably describes a subordinate group … in terms derived from its own systems of thought. These same … categories guide the dominant group’s explanations about itself. (p. xix)

We proceed, then, by elaborating upon characterizations in the women’s accounts of men’s behaviour that they said subtly disadvantaged them. In order to structure our analysis, we have loosely grouped them in interconnecting sections: ‘men support men’, ‘men don’t do support work’ and ‘men exclude women’.

### Men support men

Though it was expressed in a variety of ways, one striking aspect of many of the women’s accounts was the view that men received more favourable treatment from male managers than women did. For instance, here an interviewee observed that men did not have to abide by the same regimes as women:

I knew I wanted to influence things but I’ve had to fight tooth and nail for everything I’ve got. The men seem to get away with murder in my opinion and if I was to say and do some of the things they do, then I don’t think I’d be in the position I’m in.

Consistent with Tannen (1992), who argues that working hard does not necessarily lead to success and career progression, this woman felt that she was treated less well than her male colleagues. She thought that this dual standard was applied and not noticed by her boss, and she felt she had only made the progress she had by strict adherence to what he wanted – whereas she thought her male colleagues were not rebuked, for instance, for being late for a meeting:

[He] is very specific about time and if he’s going to meet you he’ll tell you I’ve got three minutes not five minutes not ten minutes but three minutes. If you’re late for a meeting then you’re in big trouble, me particularly, but one of the managers who is male, he can be an hour late and it’s just laughed at because it’s the norm for him.

Another woman spoke of the protection she thought a male colleague received from their joint boss:

Mike [her manager; this, and all subsequent names are pseudonyms] would never, um challenge Tom [colleague on the same grade]. You know, I think it’s because Tom’s a man, but with me and other girls in the lab as well, I think, yeah, we all get challenged if there is a problem or something. But not Tom; no, not as much; not at all.

This kind of story was told by a number of women. Indeed, the basic plot, as well as some of its discursive features (both this and the next account refer to female scientists as ‘girls’, for example), were echoed by this respondent:

Bill [a colleague] is treated [advantageously] maybe [because] he is more intimidating than a girl. I don’t know; I wouldn’t say it’s sexism except that, well, there are underlying things you couldn’t really classify as sexism. It’s all these little subtle things and it is subtle you know.

These three women believed that the cultural advantages associated with being a man were enacted in taken-for-granted and subtle ways. Like Valian (2004), they could see that men were rated more highly than they deserved, but women also shared difficulty in articulating the precise nature of the unfairness – and the reasons for it. Like one of Sonnert and Holton’s (1995) female respondents who said, ‘I don’t think I could have described well enough what I was experiencing when it was happening’ (p. 129), women in our study seemed to find it difficult to articulate their maltreatment. Such difficulties are unsurprising. Claims that men are more intimidating than women or that they are harder for managers to challenge can hardly be read off from obvious, self-evident criteria. Indeed, the interviewees’ hesitancy and caveats also suggest that they may have been conscious of their lack of power to make such claims; a lack of power that is a particularly important consideration when judgments are necessarily based on subjective criteria – and when the managers in their accounts would probably have greeted such views with scepticism, even, perhaps, with outright hostility.

In the accounts of a number of women, however, one of the most direct consequences of men’s day-to-day support for other men was in recruitment decisions. According to this respondent, for instance, men could achieve promotion for their confidence, rather than for (more conventionally defined) work-related abilities:

I’m generalizing, but on the whole, from what I’ve seen, men are able to talk a lot and to appear very confident and that seems to get them promotion, but sometimes [there is] little to back it up. One good example is Jim Brown; he has just been promoted to the same level as me because he’s a very confident person. You’d think that you’d have to have good management skills, good team building skills, as well as being a good scientist, which would be valued highly, but not here.

Thus, characteristics associated with forms of masculinity (an ability ‘to talk a lot and to appear very confident’) are believed to trump those official criteria (‘good management skills, good team building skills … being a good scientist’) that are supposedly used as the bases on which senior scientists are promoted. Indeed, such gendered readings of the ways in which suitability for promotion (really) gets assessed tend to render problematic those accounts of recruitment practices given by the men interviewed:

In some ways women are held back because they will not perform at interview; they are not fully convinced that they should be there. I’ve been amazed at some of the CVs, then I look at the person and talk to them and they do not have the confidence commensurate.

As Fotaki (2011) points out, to succeed in a man’s world, women must comply with the ‘limitations of their embodied presence – their comportment, expression, speech and so on’ (p. 48). This male Board member saw confidence as a primary reason for recruiting senior staff, and if women did not show sufficient confidence to meet his personal criteria, they were not appointed. Furthermore, in contrast to women, it also seemed to us that men, in their accounts, tended to ‘read from’ officially sanctioned scripts about individuals’ competence and merit. As one male chief executive said:

We had three women apply for the most recent recruitments to the Board, but they didn’t get it as the promotion appointments are still based on merit and competence. I think most people in [the organization] try to say when they are doing panels ‘what are the competencies you require for this job?’ and ‘who fulfils those competencies best?’

When pressed to say more precisely what constitutes the nature of criteria like ‘merit and competence’, another chief executive made particularly revealing comments, as characteristics other than ‘merit and competence’ were deemed to be more important:

We interviewed for one job, and there was a serious internal candidate, and the reason she didn’t get it was because people regarded her as too aggressive and wouldn’t make it work; [she was] very able, but divisive.

In noting the judgment that the woman was ‘very able’, but that her ability was outweighed by being considered ‘too aggressive’, we are particularly interested in what counts as ‘too aggressive’. As Tannen (2008) argues:

[i]f a woman speaks or acts in ways … expected of a woman, she will be liked but may be underestimated. If she acts in ways …expected of a person in authority, she may be respected but will probably be viewed as too aggressive. (p. 127)

The so-called aggressiveness referred to above might well be interpreted in a man as mere assertiveness or the displaying of confidence and so be welcomed as a positive quality. This example also illustrates the double standard to which women are held whereby ‘identical behaviours are not defined as the same but as different due to the sex of the performer and the social context in which they take place’ (Eichler, 1980: 16). In addition, as Eichler (1980) points out, ‘our language is tied to sex, our vocabulary is not adequate to describe the absence of sex differences when it occurs’ (p. 14).

Not only did many women believe that men constructed women as unsuitable for senior jobs, but also some of the men’s accounts lead us to suggest that men also supported other men indirectly – by constructing women as better suited for operational science jobs than men. Here, for example, is an account from a senior male healthcare scientist:

I think women are much more focused and they’re patient. If you give them a task they will do it; men will tend to deviate. And if someone comes along with something more interesting, men will move away from what they should be doing, and women don’t. I mean we’ve got a study going on now where we’ve got four women working and they are so focused. You go in there and say I think we should do this, or there’s a problem and we’re going to try and solve it, try this, try that, and it’s just done, you know they just do it. They come back with the results or they put it on the computer and the next thing you know you’ve got an e-mail with a pile of results on it. If you ask a man to do it he’ll faff around for ages and then he might change what you asked him to do because he thinks it’s better than what you suggested and so you have to go back and do it again. And there are things like that. And maybe I just think it’s easier to work with women, there’s less conflict. You do get more conflict working with men. They get all this peeing on the post sort of stuff.

Though presumably offered as a positive appraisal of women’s potential, we think that some of these claims are rendered more ambiguous when read in light of the women’s accounts. One example is the way the interviewee said he dealt with women, telling them: ‘I think we should do this, or there’s a problem and we’re going to try and solve it, try this, try that’. Perhaps for those on the receiving end of it, such behaviour might feel like getting regularly challenged – reminiscent of the account of her manager given by one of the respondents quoted above. Similarly, while one would hardly expect him to admit it directly, his evident dislike of conflict with men supports the beliefs that male managers are intimidated by other men. Indeed, his remark about men ‘peeing on the post’ suggests that he saw men’s behaviour as directly challenging his status as the laboratory’s top dog.

Furthermore, one might speculate, there is a positive side to the sort of male behaviour caricatured in this excerpt. Someone who thinks he can better his manager’s suggestion might make his manager uncomfortable, but might also be regarded as the sort of man who has ability to work on his own initiative, who has confidence and so on. Indeed, the caricature of the man who thinks he can do ‘better than what you suggested’ might be reminiscent of the Jim Brown mentioned in one of the women’s excerpts earlier – who was said to have been promoted because of his confidence. Conversely, the supposedly positive caricature of women – as cooperative – hardly has value in career terms: doing as you are told does not make you a credible candidate for senior posts. In fact, being cooperative might be interpreted as acting in relational ways; ways that ‘get disappeared’ as Fletcher (2001) memorably puts it: ‘certain behaviours “get disappeared”, not because they are ineffective but because they get associated with the feminine, relational, or so called softer side of organizational practice’ (p. 3).

In other words, it seems to us that women are caught in a no-win situation, as Tannen (2008) describes, ‘[w]omen are subject to a double bind, a damned-if-you-do and damned-if-you-don’t’ (p. 126). If they challenge managers overtly, although their (masculine) scientific abilities might be appreciated as a consequence, in failing to conform to wider cultural expectations about how women should behave, they also risk being judged too aggressive. Women are expected to behave in ways befitting women, and if they do not, they suffer for it (Adler, 1993; Bendl, 2008; Eichler, 1980; Fotaki, 2011; Tannen, 2008). Some women seemed to be aware of this double bind and found ways of coping with it, albeit not in ways they were happy about. One woman, for instance, was mindful of what she could and could not say to her boss: ‘I am very outspoken and I’m very verbal but I get on well with him because I’m submissive’. This woman realized that challenging her boss was counterproductive, so she coped by being ‘submissive’ and not questioning his requests. Her submissiveness in turn might be interpreted by her boss as a relational characteristic that was ineffective in practice (Carlson and Crawford, 2011). This may well have meant, then, that she was not given the roles that would assist her in her development as a scientist but instead was encouraged to take on the support roles that kept her in a subordinate position. Indeed, in the next section, we focus on this aspect of men’s behaviour in the women’s accounts: that they avoided taking on support roles.

### Men do not do support work

Support roles are, of course, essential for science to continue, but doing them gains one little (if any) career credit. The theme was neatly summed up in this complaint:

I’m more likely to sort out something that needs sorting than to say, ‘I’m gonna write my papers!’, whereas the chap I share an office with is very happy to say, ‘sod that to everything!’ and sit there and do nothing but write papers all year. And so, obviously, his publication record is fantastic.

Of course, this man can only write his papers all year if another scientist is prepared to take on his share of the necessary support tasks, and as Ford and Harding (2010) argue, cultural expectations surrounding gendered roles make it more likely that women will do such tasks:

[w]omen, in conforming to the appropriate configurations of gender have a higher percentage of contacts with people … spend more time … on administration work; spend more time communicating; tour buildings and care for the physical environment more. (p. 505)

Indeed, we think that the enactment of such gendered roles within general society is even stronger within science, given its historical legacy for support work to be associated with the feminine. Such a legacy acts both to devalue support work and to reinforce the cultural assumption that it is women who are the ‘natural’ support workers, who, as in nursing, tend to take on the ‘role of “handmaiden” to male professionals’ (Witz, 1990: 688). The same woman continued:

If somebody has a problem with their machine they’ll ask me rather than him. Although he has more staff to manage than me, he’s only worried about his section and doesn’t care about that except when it impacts on him.

Unsurprisingly, then, many women expressed ambivalence about doing support work. They knew it was important, may even have liked doing it, but realized that taking it on was unwise in career terms. For instance, another woman was similarly willing to take on unpopular jobs in the laboratory, but knew herself to be disadvantaged because her work was not recognized:

I’m very organized and I’ve taken on a lot of other roles within the laboratory because we’re going for accreditation at the moment and I’ve done a huge amount of work towards that [extra set of responsibilities listed], those are all the roles I do in my lab and I’m not trying to boast but I do take stuff on that I don’t have to. I do feel undervalued and I don’t think my achievements are seen.

Thus, this woman’s contribution to the smooth running of the laboratory was taken for granted and added little in terms of the recognition of her scientific advancement. Even women who were regarded as good scientists took on additional unwanted roles to support their male bosses:

I was doing what Philip wanted me to do at that time–mainly his grants and doing all his dirty work–whilst running the laboratory.

This woman complied with her boss’s bidding; she accepted the additional responsibilities, which may even have been given to her as a development opportunity but were not presented as such, and she was expected to juggle these with her existing responsibilities of running a laboratory. Given such cultural pressures on their performances as women, as Eichler (1980) describes, it is perhaps unsurprising that even established female scientists could succumb to being manoeuvred out of science in favour of support roles. One such woman described how she had been overlooked for promotion, explaining that she had been moved away from her specialist field into project management:

I was an expert in my field, and used to attend a lot of overseas committee meetings representing the UK. But, over the years, because I’ve moved away from my science subject and the overseas things and have more skills now in project-management and setting up new things, and I think that’s where they see my skills.

Like the majority of the women interviewed, this woman implied a lack of control, even assigning her career’s direction to managers: ‘that’s where *they* see my skills’. It seems that in doing project management, this woman was moved away from her scientific career, thereby becoming invisible as a scientist.

The notion that men do not do support work was indirectly commented upon by several of the men interviewed. One example came from a senior scientist who illustrated how he expected women, rather than men, to undertake support work by making the observation that women were more suited to microscope work because they had more ‘patience’ to sit ‘for several hours’:

The type of work that we’ve done for years in this lab is more suited to the female than to other … *(first author’s interjection at pause: In what way?)* well patience sitting at a microscope for several hours. Over the years we’ve tended to employ women but these are sort of technician grades at various levels for that particular type of work.

He used the word ‘technician’ in a manner that struck us as derogatory: he seemed to imply, in our reading, that women were merely employed to carry out low-grade tasks and had little potential for anything more demanding. In other words, his statements show how he worked within Valian’s (2004) ‘gender schemas’ in science, where he would ‘underrate’ women’s ability and in so doing exclude them from positions of influence (p. 208; see also Miller, 1986). It is these sorts of exclusions that are the theme of the next section.

### Men exclude women

Perhaps women’s accounts of being prepared to take on support roles are a specific example of a more general theme – that of being excluded from the work science elites have defined as important. Indeed, the final aspects of the women’s accounts we consider are stories of how women feel they get excluded from decision-making forums. Take, for example, this account from a post-doctorate researcher in her late twenties:

My boss is absolutely fantastic; he’s lovely, um; but he’s very close to the chap who was working on this project before and things tend to get decided between the two of them. I wouldn’t say it’s because I’m female I would say it’s more kind of age discrimination in a way.

More senior women’s accounts of exclusion tended to be concerned with being excluded from managerial decisions. Often, such women felt that scientific and managerial skills were not enough, in themselves, to move them into the inner circle. For instance, a female scientist, one level below the management team, commented on the significance of the absence of any women on it:

I don’t think the chief executive is very good at involving women. It’s strange, I thought I was comfortable with him, but he has surrounded himself by men. He is open in a lot of ways, but there is not a single woman in the group that is close to him.

These two accounts of being excluded (though one explicitly denied a gender dimension) are linked by a belief that men like to be ‘close’ to other men. Note, in particular, how the second respondent sees the exclusively male membership of ‘the group that is close to him’ as a feature the chief executive deliberately chose, and furthermore, it was not because women were unavailable–rather she believed the chief executive had difficulty relating to women on a professional level:

They are all men at the top and I don’t think the CEO includes women … I think women are very good managers from what I have seen but not many get up to the top.

To put the issue of male closeness in more conceptual terms, perhaps these respondents’ views of male closeness resonate with what Kanter (1977) calls male ‘homosocial reproduction’ (p. 63). Writing in the context of managerial executives, Kanter (1977) argues:

[k]eeping … [top] positions in the hands of people of one’s [own] kind provides reinforcement for the belief that people like oneself actually deserve to have such authority. ‘Homosocial’ … reproduction provide[s] an important form of reassurance in the face of uncertainty about performance measurement in high-reward, high-prestige positions. So [top] positions … become easily closed to people who are ‘different’. (pp. 62–63)

The comments from women, together with Kanter’s concept of homosocial reproduction, have informed our reading of the following male chief executive’s reflections:

We’re a small Board, but it’s not, it’s certainly not deliberate policy, and I think that … during my time here, more and more women come through to head of department level … there are a number of potential candidates for Board level among the female community; I mean I generally don’t consider it an issue, but I’m … very conscious of the fact that people look at us and say, ‘they are all men’.

Though he claimed that he did not consider it an issue, in our reading of his comments, he was clearly uncomfortable with admitting the Board’s all-male composition, even if the (unidentified) ‘people [who] look at us and say they are all men’ did not have the power to change the situation. But what we find particularly interesting in the light of the last women’s comments about her manager’s failure to involve women is the phrase ‘the female community’. Its use here almost seems to suggest that he constructed women as if they were a separate tribe from whose interests, values, emotions and so on, he was radically separate. Moreover, in speaking of the ‘female community’, he also suggested a belief in this community’s inherent inferiority; there is, after all, no explicit mention of a male community (although his Board might have been represented in exactly such terms!).

Talk of the female community suggests, therefore, that masculine practices are the unexamined norm, in line with Hearn et al.’s (2009) comment that ‘[m]anagement overwhelmingly remains men’s arenas … with clear structural gendered hierarchies’ (p. 42). Thus, this man’s talk of the ‘female community’ represents, for us, an example of the way in which women tend to get discursively constructed as a problematic ‘other’ within healthcare science, and their status as such consequently comes to legitimate and reinforce women’s exclusion from (male) communities of power. Resonating with Bendl’s (2008) description that the ‘gendered subtext … highlights the dichotomy of “male” as the norm and female as “the other”’ (p. S54), perhaps this chief executive excluded women from his board by ‘reinforc[ing] the status quo and the continued exclusion of women and other stakeholder groups from positions of power’ (p. S57). He (perhaps unknowingly) uses language with a rhetoric of inclusion ‘in order to make the board appear gender neutral’ (2008: S57) and to imply support for the advancement of women, while simultaneously encouraging an agenda of exclusion. As Hearn (1998) points out, the ‘taken-for-grantedness of men is reaffirmed through the absence of men. Men are unspoken and so reaffirmed’ (p. 787).

It is unsurprising then that one senior woman scientist commented that she had to behave carefully with a boss who had a different set of values:

There isn’t a safe environment where I can express my ideas and not get shouted down and made to feel as though I’ve said something ludicrous. I wasn’t invited to those meetings again … it was probably the wrong way to handle it but I give loyalty to my boss and follow as well as I can. I think I’m truthful and honest and I expect everybody to play by the rules that I’ve got but most of the time the men abide by one set of rules and we have another.

This woman echoed Fotaki’s (2011) comments on the ‘unrepresented woman’ where the ‘masculine logos [is] the dominant form of speech’ and where women may be ‘demolished’ in a public forum where ‘would be participants may … be silenced’ (pp. 46–47). The woman was frustrated that she could not contribute as an equal and remained excluded. In addition, it appeared that she felt that she was the one at fault, noticing her difference and assuming her deficit.

Another woman similarly commented on her lack of respect for her male boss saying he was poor at communicating and failed to involve her. Her relational way of working is important to her too:

You have to bring your team with you – I rely on my staff to help me and I think it’s a two-way thing and they know that I will support them.

Her working approach was in contrast to that of her boss who did not include her in his decision-making:

I don’t think I’ve learned anything from my own head of department. He is not good at communicating and he does not work in the same way as I do. I see decisions made behind closed doors. I’m probably not one of his favourites but I’m good at my job so he can’t get rid of me.

Her exclusion is in line with the proposal from Carlson and Crawford (2011) that women are excluded because they act in a relational way viewed as ineffective. This senior woman thought that she acted with integrity and inclusiveness but her male boss excluded her; her phrase about her boss ‘not work[ing] in the same way as I do’ possibly indicated his rejection of her difference. Furthermore, her relational way of working with her own staff was in direct opposition to his way of working with her, and we interpret this as an unspoken explanation for why he excluded her from discussions, which affected her and her team. Her bitterness at the way her boss treated her is expressed by her believing that he would prefer it if she did not report to him.

## Discussion and conclusion

In summary, the accounts from the participants in our research indicate continuing subtle forms of discrimination that largely go unnoticed – by both women and men alike in healthcare science. Our major overall finding, then, is that whatever way women act and behave within healthcare science, the discourses constituting masculinity and femininity typically reinforce interpretations that men are more suited to high-level posts than women.

We noted that women commented on how men seemingly overrated and supported other men (Valian, 2004), while men were also thought subtly to treat women as though they were inferior to men. However, it is apparent from the interviews that women rarely challenge male behaviour they see as problematic, finding ways to cope that typically reinforce their subordination. Furthermore, the senior men interviewed often identified with other males on their way up the career ladder in homosocial reproduction (Kanter, 1977) and used their image of idealized men as the gold standard by which to measure women. Women tend to be found wanting, for instance, in confidence, which is valued more highly than relational skills often regarded as ineffective.

Women are expected to undertake roles that are institutionally associated with women (the necessary laboratory support, operational work or relational activities) but these do not earn them accolades in science. Their undertaking these roles is expected not only by the male bosses but also by women themselves. However, these roles do not help women advance, and they may well contribute to women being kept in their place and not being encouraged to advance. Even when women are praised for their scientific skills, it is because they work well under direction. Some women are also moved away from their scientific specialty into management or support roles, seemingly having little control over the direction of their supposed careers. Women, then, become invisible as scientists and become excluded by men from decision-making forums. These practices remained more or less unexamined by all the healthcare scientists interviewed (male and female). In an environment in which men are constructed as better at fulfilling the requirements of top jobs, rationalizations of decisions about why more men than women still get appointed remain plausible.

These major findings resonate with other similar feminist work within science and in related disciplines. Like Rhoton (2011: 712), for example, we have shown that women accept or even collude in their own ill-treatment, and we agree that this is because they accept masculine practices as the norm in science. However, Rhoton’s (2011) interviewees ‘distinguish[ed] themselves from other women or practices and traits commonly associated with women or femininity’ (p. 701) perhaps in part because her interviewees were all senior women (‘full’, ‘assistant’ or ‘associate’ professors; p. 700) in the United States and may have needed to be competitive with everyone to progress. Women in our study did not appear to distance themselves from the practices of other women. Indeed, in our own data, we can find no disparagement of femininity per se (cf. Rhoton, 2011: 701–703). Rather, we think the so-called feminine attributes, when present in managers and colleagues, were typically welcomed by the women interviewed. We believe that there are interesting differences in healthcare science specifically that could contribute to the acceptance of other women healthcare scientists. There is a considerably larger proportion of women overall who work in healthcare science compared to SET disciplines generally, and therefore, perhaps a wider range of role models and associated discourses and behaviours. When the women interviewed commented on other women, they were supportive of them; indeed, they often saw them as good managers.

Our finding that women healthcare scientists actively value at least some characteristics culturally associated with femininity and support other women gives us a certain cause for optimism. So does the fact that while many of the interviewed women were at least initially resistant to any possibility that gender was important in their career or wider life-opportunities, as the interviews typically proceeded, there were glimpses of men’s actions thought to have had subtly negative consequences. Once women began to talk about the subtle masculinities in action that they experienced, they seemed prepared to discuss them further; it was as though articulating their disadvantage initially troubled them and talking about their experiences presented an opportunity for women to consider the men’s actions in a way they had not previously considered doing.

These findings suggest that there may be a significant number of people within healthcare science who, at least in principle, are likely to be sympathetic to feminist understandings of their situation. After all, one of us is a healthcare scientist who has been influenced by feminism; perhaps, it is not too implausible to believe that other healthcare scientists (women and men) might find that feminism has something to offer them too. Indeed, we hope that our study will provide individual healthcare scientists an opportunity to better resist the effects of subtly harmful behaviours, for example, by enabling people to see more clearly that some of their own negative experiences are not unique to them but are commonplace and significantly detrimental – making active resistance legitimate and worthwhile.

Nevertheless, we are aware of the considerable forces arrayed against feminism in healthcare science and feel that the near absence of feminist ideas from mainstream healthcare science considerations may account in part for women’s slow progression in the healthcare sector. After all, we came across no healthcare scientist who identified as a feminist – nobody, even, who articulated ideas influenced by any recognizable form of feminism. The women interviewed seemed to us to be more-or-less unaware of the mechanisms of their disadvantages, apparently accepting their lot as the way of the science world. There was no attempt, for example, to invoke the idea of ‘women’ as a collective; if anything, it was common for interviewees to do things we think would likely have the opposite effect. Women regularly called other female scientists ‘girls’, for instance; a practice that arguably risked further legitimizing and reinforcing an inferior status (Finn et al., 2010; Learmonth, 2009). Perhaps one of the problems for women who become healthcare scientists is the nature of a university science education. Most people educated in the humanities or social sciences are likely to have had some exposure to feminist thinking; science graduates, most likely, have none.

What then are the prospects for change? Some of the more recent gender-based initiatives at the policy level in science have been more sympathetic to the sorts of ideas we are advocating, giving us reasons to be guardedly optimistic. A number of recommendations from the Athena SWAN Charter Award^4^ (2010, 2011) and genSET (2010)^5^ seek improvements in areas known to be an issue for the advancement of scientists, including in recruitment practices and procedures. A best practice guide on ‘Organizational Culture’, for instance, states that the underlying assumptions of culture are the ‘unconscious, taken-for-granted beliefs, thoughts, and feelings … [and] are much harder to change’ and gives advice for mitigating their effects (Athena SWAN, 2010: no page number).

However, while the recent policy-level initiatives are to be welcomed, Tarrach (2011) comments on the genSET report:

[m]any analyses show improvement, an increase in the number of women in the upper echelons of the academic and research hierarchy. But where there is improvement it comes at too slow a rate and the evidence that it suffices for eventually solving the problem is wanting. Some people say that there is no problem; that women just do not need to dominate as men do. Maybe, but such a strong statement begs for an equally strong proof. (p. 126)

Furthermore, not all publications that would be expected to mention the importance of subtle discrimination against women do so. This leaves us considering how the subtle masculinities that pervade healthcare science and wider science workplaces will be made obvious and challenged to improve the working science environment.

We feel that the masculinist science workplace is unlikely to change much without overt pressure – especially from women. To encourage change, women (and men) have to first note the subtle forms of women’s disadvantage that they mostly currently accept as normal professional practice. Women then need to take the next step and confront the dilemmas of unfair subtle practices that are unspoken but have insidious effects and to develop the confidence to speak out.

After all, as in other walks of life, the most powerful men in healthcare science benefit from the privileges of being a man; they are therefore unlikely to question these privileges unless forced to do so (see Sismondo, 1995, for an account of this argument in a Marxist context; see also Hartsock, 1983). Unfortunately, women’s continuing relative hierarchical weakness in science – along with their relative lack of a language of contestation – means that they are doubly unlikely to be able to force senior men to question their gender privileges within science. And even if they speak out, as Kemelgor and Etzkowitz (2001) have shown, women scientists who protest overtly about gender inequality may face considerable difficulties.

For us (even though one of us has indeed faced some of the difficulties Kemelgor and Etzkowitz identified), challenging the taken-for-granted is, overwhelmingly, the more attractive option because of its potential to change things – at least in the long term. Women (and men) have a choice about whether to continue to cope with the man’s world we live in or, as feminists, ‘*behav[e] differently*’ (Stanley and Wise, 1993: 133, italics in original) and ‘render the “mundane and routine” problematic and extraordinary’ (p. 134). Indeed, we hope that this article inspires at least some of its healthcare scientist readers to behave differently, and in the future, to challenge the subtle masculinities in action. We would hope, then, that work like ours will get more of a hearing and be taken seriously within similar initiatives in the future.

## Notes

1. ‘Healthcare science’ covers the laboratory work undertaken in life sciences and pathology to aid the diagnosis and treatment of patients and includes biomedical science.

2. ‘Healthcare scientists’ in this study were qualified at least to degree level, and some had higher degrees including PhDs.

3. Subtle discrimination in various areas of science is, of course, a worldwide issue, even in countries where legislation should protect women from more blatant forms of discrimination – in the United Kingdom, the legislation is in the form of the Equality Act (2010). This is confirmed by various studies for example, in the United States, by Kemelgor and Etzkowitz (2001), Valian (2000) and Roth and Sonnert (2011) who report that ‘informal structures and anti-bureaucratic practices disadvantage both female scientists and non-scientists’ (p. 396). Similar examples are also described in France (De Cheveigné, 2009), Finland (Husu, 2001), Sweden (Peterson, 2010) and Turkey (Küskü et al., 2007).

4. In the United Kingdom, the UK Resource Centre for Women (UKRC) and the Equality Challenge Unit, together with the support from the Royal Society, fund the Athena SWAN Charter Award, which recognizes good employment practices in science, engineering and technology (SET) research institutes. Although healthcare scientists who work outside research institutes are ineligible, we find this initiative particularly encouraging because, in outlining good practice in work–life balance and employment, its principles seem to be less concentrated on the business case arguments than the UKRC itself.

5. The European Commission (EC) has been active in attempting to change policy and practice (EC, 2009; genSET, 2010) to improve the advancement of women (and men) scientists throughout Europe, again in research. The genSET report led to a special issue of the *Interdisciplinary Science Reviews* in June 2011 devoted to gender in science and taking the approach of ‘examin[ing] gender as a dimension of scientific excellence’ (Pollitzer, 2011: 101).

6. Various initiatives in the United States such as that funded by the National Science Foundation (NSF, 2010) seek to promote the advancement of women and other under-represented groups in SET careers, in particular by providing funding for institutions and projects (NSF website).

7. We were resolute in abiding by the three principles suggested by Watts (2006): ‘consent, confidentiality and [responsibility for the] conduct of the research’ (p. 386) with people who may not ‘identify with feminist aims’ (p. 385). As the interviewees did not work in the NHS, formal ethical approval was not required for the study; agreement was, however, gained from PQR’s Chief Executive.

## References

[bibr1-0306312712460606] Acker J (1990) Hierarchies, jobs, bodies: A theory of gendered organizations. Gender & Society 4(2): 139–158

[bibr2-0306312712460606] Adler NJ (1993) Competitive frontiers: Women managers in the triad. International Studies of Management & Organization 23(2): 3–23

[bibr3-0306312712460606] Albert M Laberge S Hodges BD, Regehr G Lingard L (2008) Biomedical scientists’ perceptions of the social sciences in health research. Social Science & Medicine 66(12): 2520–2531 18336978 10.1016/j.socscimed.2008.01.052

[bibr4-0306312712460606] Athena SWAN (2010) Best Practice Factsheet 1: Organizational Culture. Charter for Women in Science. Available at: http://www.athenaswan.org.uk (accessed 15 February 2012).

[bibr5-0306312712460606] Athena SWAN (2011) Measuring Success 2011. Charter for Women in Science Impact Report. Available at: http://www.athenaswan.org.uk (accessed 15 February 2012).

[bibr6-0306312712460606] Bendl R (2008) Gender subtexts – Reproduction of exclusion in organizational discourse. British Journal of Management 19: S50–S64

[bibr7-0306312712460606] Benokraitis NV Feagin JR (1995) Modern Sexism: Blatant, Subtle, and Covert Discrimination, 2nd edn. Englewood Cliffs, NJ: Prentice Hall

[bibr8-0306312712460606] Billing YD (2011) Are women in management victims of the phantom of the male norm? Gender, Work and Organization 18(3): 298–317

[bibr9-0306312712460606] Bjørnholt M (2011) How men became the local agents of change towards gender equality. Journal of Gender Studies 20(1): 3–18

[bibr10-0306312712460606] Broadbridge A Hearn J (2008) Gender and management: New directions in research and continuing patterns in practice. British Journal of Management 19(s1): S38–S49

[bibr11-0306312712460606] Calás M Smircich L (1996) From the woman’s point of view: Feminist approaches to organization studies. In: Clegg S Hardy C Nord W (eds) Handbook of Organization Studies. London: SAGE, pp. 218–257

[bibr12-0306312712460606] Carlson J Crawford M (2011) Perceptions of relational practices in the workplace. Gender, Work and Organization 18(4): 359–376

[bibr13-0306312712460606] Code L (ed.) (2000) Encyclopedia of Feminist Theories. London: Routledge

[bibr14-0306312712460606] Creager A Lunbeck E Schiebinger L (eds) (2001) Feminism in Twentieth-Century Science, Technology and Medicine. Chicago, IL: University of Chicago Press

[bibr15-0306312712460606] Crompton R Lyonette C (2011) Women’s career success and work-life adaptions in the accountancy and medical professions in Britain. Gender, Work and Organization 18(2): 231–254

[bibr16-0306312712460606] De Cheveigné S (2009) The career paths of women (and men) in French research. Social Studies of Science 39(1): 113–136 19569427 10.1177/0306312708097656

[bibr17-0306312712460606] Department of Trade and Industry (DTI) (2003) A Strategy for Women in Science, Engineering and Technology. Government Response to Set Fair, a Report from Baroness Greenfield CBE to the Secretary of State for Trade and Industry. London: DTI

[bibr18-0306312712460606] Dubois B (1983) Passionate scholarship: Notes on values, knowing and method in feminist social science. In: Bowles G Duelli Klein R (eds) Theories of Women’s Studies. London: Routledge, pp. 105–116

[bibr19-0306312712460606] Eichler M (1980) The Double Standard: A Feminist Critique of Feminist Social Science. London: Croom Helm

[bibr20-0306312712460606] Equality Act (2010) London: The Stationery Office

[bibr21-0306312712460606] European Commission (EC) (2009) The gender challenge in research funding – Assessing the European national scenes. Report no. EUR 23721 EN. Luxembourg: Office for Official Publications of the European Communities

[bibr22-0306312712460606] Finn R Learmonth M Reedy P (2010) Some unintended effects of teamwork in healthcare. Social Science & Medicine 70(8): 1148–1154 20137845 10.1016/j.socscimed.2009.12.025

[bibr23-0306312712460606] Fletcher J (2001) Disappearing Acts: Gender Power and Relational Practice at Work. Cambridge, MA and London: MIT Press

[bibr24-0306312712460606] Ford J Harding N (2010) Get back into that kitchen, woman: Management conferences and the making of the female professional worker. Gender, Work and Organization 17(5): 503–520

[bibr25-0306312712460606] Ford J Harding N Learmonth M (2012) Who is it that would make business schools more critical? A response to Tatli. British Journal of Management 23(1): 31–34

[bibr26-0306312712460606] Fotaki M (2011) The sublime object of desire (for knowledge): Sexuality at work in business and management schools in England. British Journal of Management 22(1): 42–53

[bibr27-0306312712460606] Gatrell C (2006) Interviewing fathers – feminist dilemmas in fieldwork. Journal of Gender Studies 15(3): 237–251

[bibr28-0306312712460606] genSET (2010) Recommendations for action on the gender dimension in science. Consensus Seminar Report. Available at: http://www.genderinscience.org (accessed 15 February 2012).

[bibr29-0306312712460606] Greenfield S Peters J Lane N Rees T Samuels G (2002) Set Fair: A Report on Women in Science, Engineering, and Technology from the Baroness Greenfield to the Secretary of State for Trade and Industry. London: DTI

[bibr30-0306312712460606] Harding S (1991) Whose Science? Whose Knowledge? Buckingham: Oxford University Press

[bibr31-0306312712460606] Hartsock N (1983) The feminist standpoint: Developing a ground for a specifically feminist historical materialism. In: Harding S Hintikka M (eds) Feminist Perspectives on Epistemology, Metaphysics, Methodology and Philosophy of Science. Dordrecht: Reidel, pp. 283–310

[bibr32-0306312712460606] Hearn J (1998) Theorizing men and men’s theorizing: Men’s discursive practices in theorizing men. Theory and Society 27(6): 781–816

[bibr33-0306312712460606] Hearn J (2000) Men, (pro-)feminism, organizing and organizations. Finnish Journal of Business Economics 3: 350–372

[bibr34-0306312712460606] Hearn J (2002) Alternative conceptualizations and theoretical perspectives in identities and organizational culture: A personal review of research on men in organizations. In: Aaltio I Mills A (eds) Gender, Identity and the Culture of Organizations. London: Routledge, pp. 39–56

[bibr35-0306312712460606] Hearn J Piekkari R Jyrkinen M (2009) Managers Talk about Gender: What Managers in Large Transnational Corporations Say about Gender Policies, Structure and Practices. Helsinki: Hanken School of Economics Research Reports

[bibr36-0306312712460606] Husu L (2001) Sexism, Support and Survival in Academia. Available at: http://www.nikk.no/Sexism%2C+Support+and+Survival+in+Academia.9UFRrKXx.ips (accessed 19 July 2012).

[bibr37-0306312712460606] Jeanes E Knights D Martin PY (2011) Handbook of Gender, Work and Organization. London: Wiley

[bibr38-0306312712460606] Kanter R (1977) Men and Women of the Corporation. New York: Basic Books

[bibr39-0306312712460606] Kantola J (2008) ‘Why do all the women disappear?’ Gendering processes in a political science department. Gender, Work and Organization 15(2): 202–225

[bibr40-0306312712460606] Katila S Meriläinen S (1999) A serious researcher or just another nice girl? Doing gender in a male-dominated scientific community. Gender, Work and Organization 6(3): 163–173

[bibr41-0306312712460606] Keller E (1985) Reflections on Gender and Science. New Haven, CT and London: Yale University Press

[bibr42-0306312712460606] Keller E Longino H (eds) (1996) Feminism and Science. Oxford: Oxford University Press

[bibr43-0306312712460606] Kemelgor C Etzkowitz H (2001) Overcoming isolation: Women’s dilemmas in American academic science. Minerva 39(2): 153–174

[bibr44-0306312712460606] Kirkup G Zalevski A Maruyama T Batool I (2010) Women and men in science, engineering and technology: the UK statistics guide 2010. Bradford: the UKRC

[bibr45-0306312712460606] Kirkup G Zalevski A Maruyama T Batool I (2010) Women and Men in Science, Engineering and Technology: The UK Statistics Guide 2010. Bradford: the UKRC

[bibr46-0306312712460606] Küskü F Őzbilgin M Őzkale L (2007) Against the tide: Gendered prejudice and disadvantage in engineering. Gender, Work and Organization 14(2): 109–129

[bibr47-0306312712460606] Learmonth M (2009) ‘Girls’ working together without ‘teams’: How to avoid the colonization of management language. Human Relations 62(12): 1887–1906

[bibr48-0306312712460606] Learmonth M Humphreys M (2012) Autoethnography and academic identity: Glimpsing business school doppelgangers. Organization 19(1): 99–117

[bibr49-0306312712460606] McMurray R (2011) The struggle to professionalize: An ethnographic account of the occupational position of advanced nurse practitioners. Human Relations 64(6): 801–822

[bibr50-0306312712460606] Martin J (1990) Deconstructing organizational taboos: The suppression of gender conflict in organizations. Organization Science 1(4): 339–359

[bibr51-0306312712460606] Martin P (2001) ‘Mobilizing masculinities’: Women’s experiences of men at work. Organization 8(4): 587–618

[bibr52-0306312712460606] Miller J (1986) Toward a New Psychology of Women. London: Penguin

[bibr53-0306312712460606] Morley L (1999) Organising Feminisms: The Micropolitics of the Academy. London: Macmillan

[bibr54-0306312712460606] Morley L (2006) Hidden transcripts: The micropolitics of gender in Commonwealth universities. Women’s Studies International Forum 29(6): 543–551

[bibr55-0306312712460606] National Science Foundation (NSF) (2010) Research on gender in science and engineering FY 2010. Available at: http://www.nsf.gov/funding/pgm_summ.jsp?pims_id=5475 (accessed 16 February 2012).

[bibr56-0306312712460606] Newman J (1995) Gender and cultural change. In: Itzin C Newman J (eds) Gender, Culture and Organizational Change: Putting Theory into Practice. London and New York: Routledge, pp. 11–29

[bibr57-0306312712460606] Oakley A (1981) Interviewing women: A contradiction in terms. In: Roberts H (ed.) Doing Feminist Research. Buckingham: Buckingham University Press, pp. 30–61

[bibr58-0306312712460606] Page M (2011) Gender mainstreaming – Hidden leadership. Gender, Work and Organization 18(3): 318–336

[bibr59-0306312712460606] Perriton L (2009) ‘We don’t want complaining women!’ A critical analysis of the business case for diversity. Management Communication Quarterly 23(2): 218–243

[bibr60-0306312712460606] Peterson H (2010) The gendered construction of technical self-confidence: Women’s negotiated positions in male dominated, technical work settings. International Journal of Gender, Science and Technology 2(1): 65–88

[bibr61-0306312712460606] Pollitzer E (2011) Why gender should be a priority for our attention in science. Interdisciplinary Science Reviews 36(2): 101–102

[bibr62-0306312712460606] Pringle R (1998) Sex and Medicine: Gender, Power and Authority in the Medical Profession. Cambridge: Cambridge University Press

[bibr63-0306312712460606] Ramazanoğlu C Holland J (2002) Feminist Methodology: *Challenges and Choices* London: SAGE

[bibr64-0306312712460606] Rhoton L (2011) Distancing as a gendered barrier: Understanding women scientists’ gender practices. Gender & Society 25(6): 696–716

[bibr65-0306312712460606] Risman BJ (2004) Gender as a social structure: Theory wrestling with activism. Gender & Society 18(4): 429–450

[bibr66-0306312712460606] Roth W Sonnert G (2011) The costs and benefits of red tape: Anti-bureaucratic structure and gender inequity in a science research organization. Social Studies of Science 41(3): 385–409 21879527 10.1177/0306312710391494

[bibr67-0306312712460606] Schiebinger L (1989) The Mind Has No Sex: Women in the Origins of Modern Science. Cambridge, MA and London: Harvard University Press 10.1126/science.246.4936.1502 17756008

[bibr68-0306312712460606] Sismondo S (1995) The scientific domains of feminist standpoints. Perspectives on Science 3(1): 49–65

[bibr69-0306312712460606] Sonnert G Holton G (1995) Who Succeeds in Science? The Gender Dimension. New York: Rutgers Press

[bibr70-0306312712460606] Stanley L Wise S (1993) Breaking Out Again: Feminist Ontology and Epistemology, 2nd edn. London and New York: Routledge and Kegan Paul

[bibr71-0306312712460606] Tannen D (1992) You Just Don’t Understand: Women and Men in Conversation. London: Virago

[bibr72-0306312712460606] Tannen D (2008) The double bind. In: Morrison S (ed.) Thirty Ways of Looking at Hillary. New York: Harper Collins, pp. 126–139

[bibr73-0306312712460606] Tarrach R (2011) An innovative appraisal of a CV. Interdisciplinary Science Reviews 36(2): 125–132

[bibr74-0306312712460606] Valian V (2000) The advancement of women in science and engineering. In: Women in the Chemical Workforce. A Workshop Report to the Chemical Sciences Roundtable. Washington, DC: National Academies Press, pp. 24–37 20669492

[bibr75-0306312712460606] Valian V (2004) Beyond gender schemas: Improving the advancement of women in academia. NWSA Journal 16(1): 207–220

[bibr76-0306312712460606] Wajcman J (1998) Managing Like a Man: Women and Men in Corporate Management. Cambridge and Oxford: Polity Press in association with Blackwell

[bibr77-0306312712460606] Watson T (1995) Rhetoric, discourse and argument in organizational sense making: A reflexive tale. Organization Studies 16(5): 805–821

[bibr78-0306312712460606] Watts J (2006) ‘The outsider within’: Dilemma of qualitative feminist research within a culture of resistance. Qualitative Research 6(3): 385–402

[bibr79-0306312712460606] West C Zimmerman DH (1987). Doing gender. Gender and Society 1: 125–151

[bibr80-0306312712460606] Witz A (1988) Patriarchal relations and patterns of sex segregation in the medical division of labour. In: Walby S (ed.) Gender Segregation at Work. Milton Keynes: Oxford University Press, pp. 74–90

[bibr81-0306312712460606] Witz A (1990) Patriarchy and professions: The gendered politics of occupational closure. Sociology 24(4): 675–690

[bibr82-0306312712460606] Witz A (1992) Professions and Patriarchy. London and New York: Routledge

